# Case Report: Extracorporeal photopheresis for BK virus nephropathy as a novel treatment for high-risk rejection kidney transplant recipient

**DOI:** 10.3389/fneph.2025.1625060

**Published:** 2025-07-21

**Authors:** Marilena Gregorini, Claudia Del Fante, Tefik Islami, Maria Antonietta Grignano, Nicoletta Serpieri, Cesare Perotti, Gianluca Viarengo, Alessia Locurcio, Giuseppe Lanotte, Alessandro Tragni, Emma Diletta Stea, Chiara Martinelli, Alessandro Marchi, Valentina Portalupi, Andreana De Mauri, Elisabetta Margiotta, Eleonora Francesca Pattonieri, Grazia Soccio, Teresa Rampino

**Affiliations:** ^1^ Unit of Nephrology, Dialysis and Transplantation, Fondazione Istituto di Ricovero e Cura a Carattere Scientifico (IRCCS) Policlinico San Matteo, Pavia, Italy; ^2^ Department of Internal Medicine and Therapeutics, University of Pavia, Pavia, Italy; ^3^ Division of Immunohaematology and Transfusion Service, Fondazione Istituto di Ricovero e Cura a Carattere Scientifico Policlinico San Matteo, Pavia, Italy

**Keywords:** ECP, kidney transplantation, antibody mediated rejection, BK virus, BKVAN, high sensitized

## Abstract

**Background:**

BK virus-associated nephropathy (BKVAN) is a major complication in kidney transplantation caused by the reactivation of latent BK virus (BKV) under immunosuppression. BKVAN has been strongly associated with increased graft loss. Currently, there is no effective antiviral treatment for BKVAN. Additionally, the development of donor-specific antibodies (DSAs) and the risk of acute and chronic rejection complicate the reduction of immunosuppressive therapy (IS). This case report illustrates the management of BKVAN in a highly sensitized transplant recipient and explores the potential use of extracorporeal photopheresis (ECP) as an immunomodulatory tool.

**Case:**

44-year-old Caucasian woman with a history of failed prior transplant and multiple transfusions underwent a second kidney transplant. Due to a high panel-reactive antibody level, she received induction therapy with plasma exchange, thymoglobulin and steroids, followed by maintenance with tacrolimus, mycophenolate mofetil (MMF), and steroids. Initial graft function was good, and protocol biopsies showed no rejection. In year four, the patient developed an increasing BKV viremia (peak of 40,050 copies/mL) and MMF was reduced, which cleared BKV in six months. Two years later, DSAs reappeared, which led to an increase in MMF. In August 2020 the patient showed a decline of GFR, elevated BKV viremia (peak 162,000 copies/mL), and a graft biopsy was performed revealing BKVAN. IS was reduced (MMF was discontinued, and tacrolimus was tapered). After eight months, the viremia cleared up, but anti-DR53 DSAs (MFI 16000) levels increased significantly. As the patient was highly sensitized and had a thrombosis of arteriovenous fistula, mTOR inhibitors were not recommended. In order to modulate alloimmunity without further suppressing antiviral immunity, ECP was introduced. Over the next two years, the patient showed stable renal function (eGFR 30–40 mL/min), no recurrence of BKV viremia, and a gradual reduction in DSAs titers. No acute rejection episodes occurred.

**Conclusions:**

In a highly sensitized patient with BKVAN and contraindications to standard therapies, ECP combined with a tailored immunosuppressive regimen proved effective in controlling viral replication, preserving graft function, and mitigating alloimmune risks. Considering the potential of ECP as an adjunctive therapy in complex BKVAN scenarios, further investigation is warranted.

## 1 Introduction

BK virus (BKV) is a common virus typically acquired in childhood (1) which establishes lifelong latency primarily in uroepithelial cells, including renal tubular epithelial and urinary tract urothelial cells (2). In immunocompetent individuals, viral replication remains suppressed, and clinically silent (3). However, in immunosuppressed hosts, such as kidney transplant recipients or patients with HIV-induced immunodeficiency, BKV may reactivate, leading to uncontrolled viral replication and potential tissue damage (4).

In kidney transplant recipients, BKV reactivation can lead to BK virus-associated nephropathy (BKVAN), a disease that significantly threatens graft function and ultimately can result in graft loss or return to dialysis (5). Among markers of infection, plasma viremia is a stronger predictor of BKVAN development and progression than viruria alone (5–7).BK viral loads exceeding 10^4^ copies/mL should raise suspicion for BK virus-associated nephropathy (BKVAN), as they are strongly associated with an increased risk of graft dysfunction. Recent data suggest that BKVAN is linked to more than a fourfold increase in the risk of graft loss (8). Despite its clinical relevance BKVAN remains a significant therapeutic challenge, as no antiviral agents with proven efficacy against BKV are currently approved.

The primary therapeutic approach remains reduction of immunosuppressive therapy aiming to restore the host’s antiviral immune response (9). However, this strategy must be carefully balanced, as excessive immunosuppression reduction increases the risk of acute or chronic rejection, potentially leading to irreversibly allograft damage (9, 10). Alternative strategies, including the use of leflunomide, cidofovir, or intravenous immunoglobulins (IVIG) has been reported in limited cases, but evidence supporting their effectiveness remains scarce (11, 12). Currently, the management focus on early diagnosis and personalized adjustment of immunosuppressive regimens, which typically includes discontinuation of antimetabolites, dose reduction of calcineurin inhibitors (CNI) and the introduction of mTOR inhibitors (13). The ideal treatment should effectively control BKV replication without increasing the risk of graft rejection, therefore, there remains a significant unmet need for novel and more effective therapeutic strategies (13).

In this contest extracorporeal Photopheresis (ECP) has emerged as a potential innovative option. ECP promotes immune tolerance and modulate immune response without inducing global immunosuppression (14). By preserving antiviral immune response, while mitigating rejection risk ECP may offer a balanced and efficacy approach to BKVAN management (15).

We report the case of a highly sensitized female patient with a history of a previous kidney transplant, who developed BK virus-associated nephropathy after a second transplant. Management was particularly challenging and ECP proved to be a valuable therapeutic adjunct, helping to strike a balance between viral control and alloimmune regulation.

## 2 Case description

A 42-year-old Caucasian woman on hemodialysis treatment for 12 years underwent a second kidney transplant. Her first transplant, performed after two years of hemodialysis treatment, was complicated by massive postoperative hemorrhage that required graft explantation after 36 hours and multiple blood transfusions. This led to significant alloimmunization, with a development of anti-DR53 antibodies that were not present in the first donor’s HLA typing and showed a fluctuating trend (DR53 antibodies consistently remained below 3000 MFI.)

This hypersensitization resulted in a 10-year waiting period before the patient could be selected as the primary recipient for a compatible organ. This allocation was made despite her history of donor-specific antibodies (DSAs, DR53) antibodies, as their titer had remained below the threshold for the past year.

For the second transplant, the patient received a kidney from a B Rh-negative donor, with whom she shared three HLA antigens (two at locus A and one at locus DR). At the time of transplant, her serology was positive for CMV, EBV, and Toxoplasma gondii but lacking data for DR53 antibodies at baseline. One pre-transplant plasma exchange (PE), followed by four additional sessions post-transplant, were scheduled for precautionary purposes, despite MFI levels being below the threshold in the last available pre-transplant serum sample.

Induction immunosuppression consisted of thymoglobulin and methylprednisolone. Maintenance therapy included tacrolimus (target trough level 10–12 ng/mL in the first three months, then reduced to 8–10 ng/mL), mycophenolate mofetil (MMF) 1g twice a day (BID), and methylprednisolone (16 mg/day in the first three months, then tapered to 4 mg/day gradually). Protocol renal biopsies at 6 and 12 months showed no histological signs of rejection. At one year post-transplant, the estimated glomerular filtration rate (eGFR) was 51 mL/min. Two years later, donor-specific antibodies (DSAs) were detected without a decline in renal function. During the following months, the patient was hospitalized for influenza A viral pneumonia, which led to acute kidney injury with a peak serum creatinine of 2.0 mg/dL. Renal function returned to baseline (creatinine 1.32 mg/dL; eGFR: 49 mL/min) at discharge. During the infection, MMF was temporarily reduced to 500 mg BID and then restored to 1 g BID after resolution.

There was no evidence of BK virus (BKV) infection during the pre-implantation protocol biopsy, performed according to the center’s clinical practice. At 9 months after transplantation, BKV viruria (276,786,000 copies/mL) was detected, associated with mild creatinine elevation (creatinine 1.2 mg/dL), and a kidney biopsy was performed to rule out viral nephropathy. The biopsy findings showed features of calcineurin inhibitor toxicity, including isometric vacuolization of tubular cells, but immunostaining for BK virus was negative.

BKV viremia did not appear until the fourth year after transplantation, when it peaked at 40,050 copies/mL. The patient’s MMF dosage was reduced to 250 mg BID, leading to negative viremia after 6 months.

Two years later, anti-DR53 DSAs reappeared (10000 MFI), necessitating an increase in MMF dose to 750 mg BID. Shortly after, the patient was hospitalized again for diarrhea and Norovirus colitis, with serum creatinine rising to 2.0 mg/dL and then stabilizing at 1.5 mg/dL. Due to poor gastrointestinal tolerance, MMF was maintained at 500 mg BID.

By August 2020, renal function had worsened (creatinine 1.9 mg/dL), DSAs reemerged (5400 MFI), and her BKV viremia increased, peaking at 162,200 copies/mL. Renal biopsy confirmed BKV-associated nephropathy, with histological findings consistent with class 2 BKVAN according to the 2018 Banff classification (16).

We reduced the immunosuppressive load by discontinuing MMF, reducing tacrolimus to minimal levels (2–4 ng/mL), and maintaining prednisone at a daily dose of 4 mg. Within six months, full viral clearance and improvement in renal function were achieved, with a stable creatinine concentration of 1.4 mg/dL.

Following a paucisymptomatic infection with SARS-CoV-2 (April 2022), the patient experienced a relapse of BKV viremia (5130 copies/mL), rising creatinine (1.9 mg/dL), and recurrence of anti-DR53 DSAs (9000 MFI). Concurrently, acute thrombosis of the arteriovenous fistula occurred. The introduction of a mTOR inhibitor, which had previously been considered, was discarded due to this thrombotic event.

**Figure 1:**
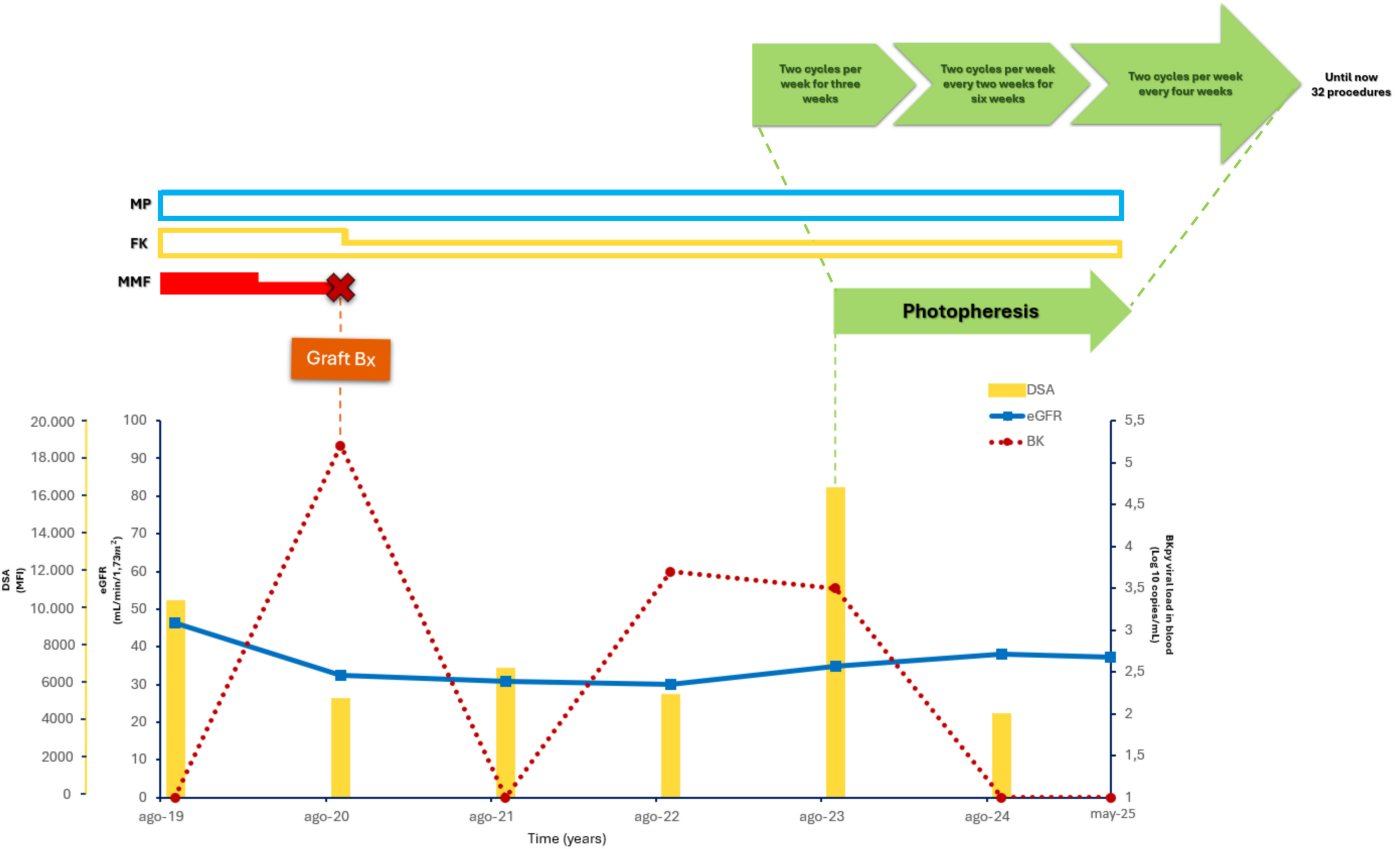
Clinical case timeline. This timeline illustrates the patient’s medical journey, detailing key treatments (FK, MP, MMF), and photopheresis cycles. The lower graph tracks DSAs, eGFR, and BK levels over time, pinpointing significant events such as the graft biopsy and variations in photopheresis schedule.

Due to the complexity of the clinical case and limited options for pharmacologic immunosuppression, ECP was initiated (**Figure 1**). The ECP treatment schedule was borrowed from the protocol used in our Centre for lung chronic rejection, and was modified as follows: 1 cycle (i.e., 2 procedures) per week for 3 weeks, 1 cycle fortnightly 2–3 times, 1 cycle per month if the patient was improved/stabilized; then, the patients were maintained chronically on ECP, progressively lengthening the treatment intervals to 2 months.

From 2023 to 2025, the patient remained free of acute rejection episodes, with stable graft function (serum creatinine 1.5–1.7 mg/dL, eGFR 35–40 mL/min), and no recurrence of BK viremia. Anti-DR53 DSAs levels fluctuated, but they showed an overall downward trend (MFI decreasing to approximately 4,000 by 2024), reflecting the effectiveness of combined immunologic monitoring, targeted ECP therapy, and tailored immunosuppression. No significant proteinuria was observed.

Over a decade after transplantation, the patient maintains stable renal function, negative viremia, and controlled DSAs levels, with no clinical signs of rejection.

## 3 Discussion

BK virus-associated nephropathy represents one of the most challenging complications of kidney transplantation, often resulting in graft dysfunction and loss (8). The mainstay of current BKVAN management is the reduction of immunosuppression to restore antiviral immune responses and promote viral clearance. However, this strategy inherently increases the risk of triggering alloimmune responses, including the development or elevation of DSAs levels, potentially leading to antibody-mediated rejection.

The present case exemplifies the complexity of this delicate immunological balance. Our patient, highly sensitized due to a prior failed transplant and multiple transfusions, underwent a second kidney transplant despite a positive virtual crossmatch and moderate HLA mismatch, which represented a high immunological risk. Seven years post-transplant, the patient developed significant BKV viremia, and biopsy confirmed BKVAN. Following standard practice, MMF was withdrawn, and tacrolimus levels were reduced. This strategy led to complete viral clearance within six months but also triggered a marked increase in anti-DR53 DSAs levels.

Standard BKVAN treatment algorithms typically recommend replacing antimetabolites with mTOR inhibitors and further tapering CNIs in such settings (17–19). In this case, however, the occurrence of acute arteriovenous fistula thrombosis made mTOR inhibitor initiation unsafe (20). Additionally, the marked increase in DSAs levels following immunosuppression reduction alone raised concerns that, even if mTOR inhibitors were a viable option, they might not be sufficient to adequately control alloimmunity in this high-risk, sensitized patient (21).

Faced with this complex clinical scenario—persistent BKV infection, histologically confirmed BKVAN, elevated DSAs, and limited options for immunosuppression (IS) adjustment—extracorporeal photopheresis was introduced.

To date, the mechanisms of action of ECP are incompletely understood. ECP may exert its immunomodulatory effects through several interrelated pathways. A central mechanism involves the induction of a tolerogenic immune environment via modulation of antigen-presenting cells, particularly through the generation of regulatory dendritic cells (DCregs).

These DCregs exhibit a diminished capacity to stimulate effector T cells and actively promote the expansion and functional enhancement of regulatory T cells (Tregs), which are critical in maintaining peripheral tolerance.

Additionally, ECP appears to influence the cytokine milieu, favoring anti-inflammatory profiles—such as increased interleukin-10 (IL-10) and transforming growth factor-beta (TGF-β)—that further support Treg differentiation and suppress pro-inflammatory Th1/Th17 responses (22–25).

This immunological reprogramming allows for a recalibration of the host immune response, promoting viral clearance by preserving or restoring virus-specific immunity while minimizing alloimmune-mediated graft injury. Unlike conventional IS therapies that broadly suppress effector T cell function, ECP offers a more targeted immunoregulatory effect, enabling reductions in the pharmacologic IS burden. This may not only enhance antiviral immune surveillance but also reduce the risk of opportunistic infections and other IS-related toxicities.

This immunological rationale is further supported by encouraging prior reports (15).

Notably, a combined lung-kidney transplant recipient with severe BKV viremia and BKVAN achieved complete viral clearance and maintained graft stability under ECP and IS minimization (tacrolimus dose reduction, MMF withdrawal), with no evidence of rejection over one year of follow-up. This suggests that ECP can act as a “facilitator” of IS minimization in high-risk settings. Additionally, a small case series reported successful control of BKV and CMV infections in kidney transplant recipients who received ECP as adjunctive therapy during rejection management (14).

In our case, the initiation of ECP alongside a carefully titrated IS regimen (low-dose tacrolimus and methylprednisolone) yielded sustained viral clearance, preserved renal graft function across a two-year follow-up, and a progressive decline in anti-DR53 DSAs levels. Remarkably, no episodes of acute rejection occurred. These favorable outcomes support the hypothesis that ECP, combined with individualized IS management, can enable simultaneous control of viral replication and alloimmune responses in patients for whom standard options are contraindicated. The observed DSAs reduction aligns with evidence from other clinical contexts, such as chronic ABMR, where ECP has demonstrated immunomodulatory efficacy (26).

In conclusion, this case highlights the challenges of managing BKVAN in the context of complex immunological profiles and therapeutic contraindications, emphasizing the limited availability of effective treatment options.

It suggests that ECP, when integrated into a personalized immunosuppressive regimen, may represent a valuable adjunct or alternative in selected high-risk kidney transplant recipients with BKVAN, offering a dual benefit: effective viral control and mitigation of alloimmune injury.

While prospective studies are warranted to elucidate the full spectrum and durability of ECP’s immunologic effects in the transplant setting, our clinical experience highlights its potential as a non-conventional but promising therapeutic option.

DSAs levels (yellow bars), eGFR (blue line), BKV viral load in plasma (red dotted line), ECP (green arrow), IS treatment (MMF (red bar), FK (yellow bar), MP (light blue bar)

## Data Availability

The original contributions presented in the study are included in the article/supplementary material. Further inquiries can be directed to the corresponding author.
